# The relative importance of perceived doctor’s attitude on the decision to consult for symptomatic osteoarthritis: a choice-based conjoint analysis study

**DOI:** 10.1136/bmjopen-2015-009625

**Published:** 2015-10-26

**Authors:** Domenica Coxon, Martin Frisher, Clare Jinks, Kelvin Jordan, Zoe Paskins, George Peat

**Affiliations:** 1 Arthritis Research UK Primary Care Centre, Research Institute for Primary Care & Health Sciences, Keele University, Keele, UK; 2 Centre for Population Health Sciences, The University of Edinburgh, Edinburgh, UK; 3 The School of Pharmacy, Keele University, Keele, UK

**Keywords:** PRIMARY CARE, osteoarthritis

## Abstract

**Objectives:**

Some patients spend years with painful osteoarthritis without consulting for it, including times when they are experiencing persistent severe pain and disability. Beliefs about osteoarthritis and what primary care has to offer may influence the decision to consult but their relative importance has seldom been quantified. We sought to investigate the relative importance of perceived service-related and clinical need attributes in the decision to consult a primary care physician for painful osteoarthritis.

**Design:**

Partial-profile choice-based conjoint analysis study, using a self-complete questionnaire containing 10 choice tasks, each presenting two scenarios based on a combination of three out of six selected attributes.

**Setting:**

General population.

**Participants:**

Adults aged 50 years and over with hip, knee or hand pain registered with four UK general practices.

**Outcome measures:**

Relative importance of pain characteristics, level of disruption to everyday life, extent of comorbidity, assessment, management, perceived general practitioner (GP) attitude.

**Results:**

863 (74%) people responded (55% female; mean age 70 years, range: 58–93). The most important determinants of the patient's decision to consult the GP for joint pain were the extent to which pain disrupted everyday life (‘most’ vs ‘none’: relative importance 31%) and perceived GP attitude (‘legitimate problem, requires treatment’ vs ‘part of the normal ageing process that one just has to accept’: 24%). Thoroughness of assessment (14%), management options offered (13%), comorbidity (13%) and pain characteristics (5%) were less strongly associated with the decision to consult.

**Conclusions:**

Anticipating that the GP will regard joint pain as ‘part of the normal ageing process that one just has to accept’ is a strong disincentive to seeking help, potentially outweighing other aspects of quality of care. Alongside the recognition and management of disrupted function, an important goal of each primary care consultation for osteoarthritis should be to avoid imparting or reinforcing this perception.

**Strengths and limitations of this study**

- Unlike many previous studies of what determines the decision (not) to consult for painful osteoarthritis, by using a conjoint analysis design we were able to quantify the relative importance of both service-related factors and patient/problem characteristics.
- Our study was large, recruited participants across a wide spectrum of characteristics and severity, had a high response rate and involved members of the public in the design stages through a series of meetings and qualitative developmental studies.
- The preference for pen-and-paper administration and the complex nature of the attributes meant that we could only include six potentially important determinants of the decision to consult and we were unable to estimate precisely the effect of interactions between determinants.

## Introduction

Osteoarthritis has a substantial and growing impact on population health,^1^ ^2^ health services^3^ and economies worldwide.^4^ Rising rates of primary hip and knee arthroplasty^5^ and projected future increases in prevalence driven by changes in population age structure and in rates of obesity and sedentary behaviour have prompted increasing calls for greater emphasis on prevention and control^6^ and ‘concerted public health and high-quality integrated medical care’.^7^ Yet it appears that some patients may spend years with painful osteoarthritis without consulting for their joint problem,^8–11^ including times when they are experiencing persistent severe pain and disability.^12^ ^13^ This is despite the fact that most such individuals will continue to consult for other comorbid conditions and that there are a wide range of recommended non-surgical management options.^14–16^ Understanding what influences the decision to consult primary care is therefore important for identifying barriers to meeting the needs of patients with this common chronic condition.

There is a wealth of studies on the determinants of healthcare utilisation in general.^17^ ^18^ Studies specifically designed to reveal the determinants of primary care consultation for joint pain and osteoarthritis have been of broadly two types: quantitative observational studies comparing the particular characteristics of consulters and non-consulters and qualitative studies on osteoarthritis patients’ experiences of primary healthcare and their reasons for seeking help.^19^ While the degree of disruption to daily activities emerges fairly consistently as a need-related determinant of consultation, qualitative studies have identified several potentially powerful beliefs about osteoarthritis and what primary care has to offer. They include beliefs and expectations on adequate clinical assessment,^20^ the perception of a limited repertoire of effective treatments,^21^ ^22^ the attribution of symptoms to ‘normal ageing’,^13^ ^23^ the importance of judging symptoms as ‘unusual’^22^ and competing priorities from comorbid illness.^24^ These may vary within individuals over time and many are likely to be shared by health professionals and patients alike. However, their relative importance has seldom been quantified.^25^ ^26^

Therefore, the aim of our study was to quantitatively estimate the relative importance of some of these perceptions of osteoarthritis primary care against established need-related factors on patients’ willingness to visit the doctor. To achieve this we undertook a conjoint analysis study in a community sample of adults aged 50 years and over with peripheral joint pain.

## Methods

### Overview of design

The design was a partial-profile choice-based conjoint analysis study, administered as a single postal self-complete questionnaire that was mailed in February 2011 to 1170 adults aged over 50 years with hip, knee or hand pain and registered with one of four general practices in North Staffordshire, UK. Participants were members of an existing population observational cohort intended to describe and predict the long-term course of joint pain and osteoarthritis—the North Staffordshire Osteoarthritis Project (NorStOP1 & NorStOP2).^27^ ^28^ The NorStOP cohorts were formed in 2002–2003 with a census survey (two-stage postal questionnaire) of all adults aged 50 years and over registered with participating general practices. To be eligible for inclusion in the current study, NorStOP cohort members had to have consented to further contact at baseline, 3 and 6-year follow-up, have reported hip, knee or hand pain in the past 12 months at 6-year follow-up, still be alive and registered with the practice at the time of mailout, and not be currently participating in other research studies in the Institute. The list of potentially eligible cohort members was then screened by the lead general practitioner (GP) at each practice to exclude vulnerable groups, for example, new-onset dementia or severe/terminal illness. Conjoint analysis and discrete choice experiments cover a range of quantitative methods for eliciting preferences and have been used previously to elicit patients’ preferences for access, content, style and provider of UK primary care consultations,^29–35^ out-of-hours care^36^ ^37^ and knee osteoarthritis patients’ and practitioners’ preferences for treatment.^38–43^ In conjoint analysis, respondents’ preferences or values for various health states or services are elicited over a range of attributes and levels that define profiles in a series of choice tasks.^44^ Our study, including the selection of attributes and levels to characterise relevant profiles, was designed with specific reference to guidance on good research practices for conjoint analysis,^44^ other key methodology sources^45–47^ and with close patient/public involvement via our Institute's Research User Group (RUG).^48^ The RUG, originally formed in 2006, was established as dedicated infrastructure to support strong patient and public involvement (PPI) to ensure that our research leads to improvements in health policy, clinical practice and patient benefit. It now comprises over 75 members with a dedicated Coordinator and Support Assistant. Members of the RUG collaborate with researchers to maintain a focus on the patient perspective through their contributions to formulating research questions, advising on methods (questionnaire design, recruitment and consent procedures), interpreting findings and advising on dissemination strategies.

### Design of questionnaire and choice task

We selected and specified salient attributes, levels and profiles based on the following main sources: (1) a narrative review of published studies of the determinants of primary care consultation for joint pain or osteoarthritis;^9^ ^12^ ^13^ ^25^ ^26^ ^49–58^ (2) a review of 15 previous conjoint analysis studies of, or including, aspects of the primary care consultation,^29–37^ ^59–64^ (3) cognitive interviews with three RUG members aged 50 years with experience of long-term joint pain and focused discussion groups with 10 RUG members. From these sources, and being mindful of RUG members’ consistent preference for simple pen-and-paper format and their concern to minimise respondent burden, we selected three clinical need-related attributes and three service-related attributes (table 1). RUG members checked the phrasing of attribute levels for comprehension. The two 2-level attributes and four 3-level attributes created 324 possible scenarios.

**Table 1:** Attributes and levels included in choice tasks

Attributes		Levels
1	Pain characteristics	You are experiencing a dull aching pain, which is there most of the time
		You are experiencing short episodes of more severe, often unpredictable pain
2	Level of disruption to everyday life	The pain is not disrupting your everyday life
		The pain is disrupting some of your everyday life
		The pain is disrupting most of your everyday life
3	Comorbidity	You are experiencing no other physical health problems
		You are experiencing other minor physical health problems
		You are experiencing other major physical health problems
4	Assessment	The GP asks about your symptoms and their effect on your day-to-day life
		The GP conducts a thorough physical examination of the joints as well as asks about your symptoms and their effect on your day-to-day life
		The GP investigates with appropriate X-rays and blood tests as well as asks about your symptoms and their effect on your day-to-day life and conducts a thorough physical examination of the joints
5	Management	The GP prescribes pain relief and gives verbal advice about your condition
		The GP prescribes pain relief, gives written advice about your condition and arranges follow-up with a practice nurse and physiotherapy referral
		The GP offers a promising new treatment as well as prescribing pain relief, giving written advice about your condition and arranging follow-up with a practice nurse and physiotherapy referral
6	GP attitude	The GP regards your joint pain as part of the normal ageing process that one just has to accept
		The GP regards your joint pain as a legitimate health problem that requires treatment

GP, general practitioner.

**Figure 1:**
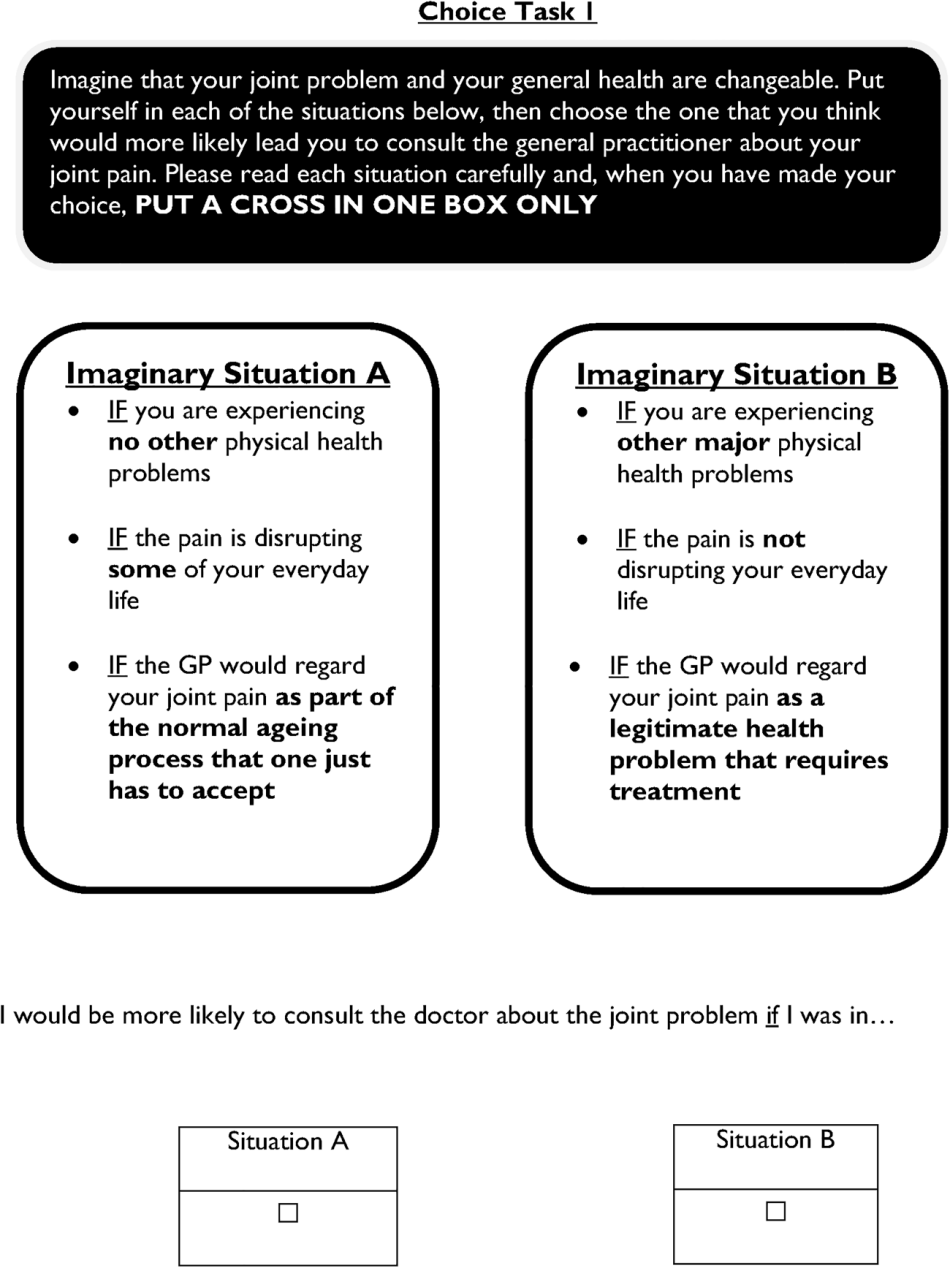
Sample page in the questionnaire showing the choice task format. GP, general practitioner.

We used pairwise choice sets: for each choice set respondents were presented with two alternative scenarios and invited to indicate under which they would be more likely to go to the GP. Owing to the relatively complex attributes and levels in this study, RUG members felt that scenarios with more than three attributes to consider in each choice task would be cognitively burdensome. We therefore chose a partial-profile design, randomly rotating attribute levels into the choice sets, such that across all choice sets each respondent would still typically consider all attributes and levels^65^ (sample choice task in figure 1).

We used the Advanced Design Module within Sawtooth Software SSI Web (V.7.0) to evaluate the relative statistical efficiency by simulating different numbers of choice sets and questionnaire versions. The combination of 10 choice sets per participant and 10 questionnaire versions based on a conservative estimate of 400 respondents (<40% response) offered acceptably precise estimates of main effects (SEs <0.05).

In addition to the 10 choice sets, the 26-page survey questionnaire included one closed question on the perceived difficulty of the choice tasks (response options: not at all hard, a little hard, quite hard, very hard, extremely hard) and sections on joint pain (previous history, recent healthcare use, Brief Illness Perceptions Questionnaire: Revised,^66^ and basic sociodemographic characteristics).

### Survey administration

The survey was administered using a standard 3-stage mailing procedure with initial mailout of questionnaire and patient information sheet. Non-respondents were sent a reminder postcard at 2 weeks, followed by a repeat questionnaire at 4 weeks.

### Statistical analysis

We analysed responses to the choice tasks by multinomial logistic regression using the aggregate logit function in CBC for SSI Web software (Sawtooth Software, Inc, Orem, Utah, USA). We estimated standardised utilities and SEs for each of the attribute levels. These utilities are zero-centred within each attribute and thus the values are relative, not absolute—for example, a negative utility value is interpreted as meaning only that this attribute level was associated with a lower likelihood of consulting relative to the other levels of that attribute. We then calculated the relative importance of each attribute as the range in utility estimates within an attribute divided by the sum of the ranges in utility estimates for all attributes, expressed as a percentage.^67^ This measure of the relative importance of each attribute is study-specific (ie, must be interpreted in the context of the attributes in the model and the levels of those attributes).

We looked at specific scenarios in which the overall utilities of two paired profiles were directly compared to predict which profile was more likely to lead to general practice consultation. The higher the overall utility of the profile, the greater is the relative propensity to consult. The utilities can be used to estimate strengths of preference for each profile, and results are accumulated over respondents to provide shares of preference among scenarios. The profile utilities are exponentiated and shares are normalised to sum to 100%.

## Results

### Response and descriptive characteristics

Of 1170 mailed, 10 were subsequently excluded having either recently died, left the practice or were no longer at the address. A further 297 potential participants refused or did not respond, leaving 863 respondents (mean age 70 years (SD 7.5); 55% female; response rate 74%.^68^ The descriptive characteristics of respondents are provided in table 2. Respondents were younger than non-respondents/refusals but did not differ on other measured sociodemographic factors (see online supplementary material file).

**Table 2:** Descriptive characteristics of respondents

	Respondents (n=863)
Age (years)	
50–64	245 (28)
65–74	367 (43)
75+	251 (29)
Female	478 (55)
Lives alone	185 (21)
Currently in full-time or part-time paid employment	196 (24)
Occupational class*	
Higher managerial, administrative and professional occupations	203 (25)
Intermediate occupations	190 (23)
Routine and manual occupations	418 (52)
Perceived financial strain: ‘quite comfortably off’†	150 (17)
Self-rated health: fair/poor	224 (26)
Number of self-reported comorbidities (0–22): median (IQR)	4 (2, 6)
HADS (0–21): median (IQR)	
Anxiety	5 (2.5, 8)
Depression	3 (1, 6)
Hip pain in past 12 months	483 (56)
Knee pain in past 12 months	633 (73)
Hand pain in past 12 months	589 (68)
Time since onset of joint problem (years)	
<1	32 (4)
1–5	243 (28)
6–10	241 (28)
>10	336 (39)
Never consulted GP for joint problem	141 (16)
Consulted GP for joint problem in past 12 months	434 (50)

Figures are numbers (percentage) of respondents unless otherwise stated.

*Standard occupational classification based on current/last job title.^71^ ^72^

†From Thomas.^70^

GP, general practitioner; HADS, Hospital Anxiety and Depression Scale.^69^

#### Relative importance of attributes

The choice tasks were well-completed (<5% missing) with most respondents rating them as ‘not at all hard’ or ‘a little hard’. Table 3 illustrates the standardised, zero-centred partworth utilities for all attribute levels and the attribute utility ranges, which form the basis for quantifying the relative importance of the attributes. The level of disruption to everyday life had the highest relative importance on the decision to consult (31%), followed by GP attitude (24%).

**Table 3:** Perceived importance of attributes and levels from choice tasks

Attributes and levels		Number of times selected/number of times presented (%)	Standardised utility (β) (95% CI)	Attribute utility range	Attribute importance scores (%)*
1. Pain characteristics					
	Dull ache	2040/4315 (47)	−0.08 (−0.114 to −0.048)	0.16	5
	Severe unpredictable episodes	2275/4315 (53)	0.08 (0.048 to 0.114)		
2. Level of disruption to everyday life					
	None	835/2840 (29)	−0.65 (−0.706 to −0.590)	1.10	31
	Some	1590/2859 (56)	0.20 (0.147 to 0.255)		
	Most	1892/2931 (65)	0.45 (0.392 to 0.502)		
3. Comorbidity					
	None	1275/2944 (43)	−0.22 (−0.276 to −0.169)	0.46	13
	Minor	1390/2836 (49)	−0.01 (−0.065 to 0.044)		
	Major	1651/2850 (58)	0.23 (0.179 to 0.287)		
4. Assessment					
	Asks about symptoms and impact	1136/2853 (40)	−0.27 (−0.328 to −0.219)	0.48	14
	As above plus thorough physical examination	1507/2842 (53)	0.06 (0.007 to 0.117)		
	As above, appropriate X-rays /bloods	1678/2935 (57)	0.21 (0.157 to 0.265)		
5. Management					
	Pain relief, verbal advice	1178/2930 (40)	−0.26 (−0.312 to −0.208)	0.45	13
	Pain relief, written advice, PN f/up, PT referral	1530/2858 (54)	0.07 (0.012 to 0.120)		
	Pain relief, written advice, PN follow-up, PT referral, promising new treatment	1609/2842 (57)	0.19 (0.141 to 0.247)		
6. GP attitude					
	Normal ageing, accept it	1360/4315 (32)	−0.43 (−0.466 to −0.397)	0.86	24
	Legitimate problem, requires treatment	2955/4315 (68)	0.43 (0.397 to 0.466)		

*Attribute utility range/sum total of attribute utility ranges.

GP, general practitioner; PN f/up, practice nurse follow-up; PT physiotherapy.

#### Pairwise scenarios

##### Proposition 1: Changing to a (GP with a) positive legitimising attitude would precipitate the presentation of less disabling joint problems

The pairwise analysis in table 4 suggests that, assuming all other factors are equal, 65% of respondents would rather consult with a joint problem that was causing some disruption to their everyday life if the GP was expected to have a ‘legitimising’ attitude (scenario A) than consult if their joint problem that was causing greater disruption to their everyday life but they expected the GP to have a ‘normal ageing-accept it’ attitude (scenario B).

**Table 4:** Paired analysis to evaluate specific hypotheses

Profile	Attributes and levels	Total utility	Probability of choosing profile* (%)
Scenario 1: *To what extent would changing to a (GP with a) positive legitimising attitude precipitate the presentation of less disabling joint problems?*			
A	The pain is disrupting most of your everyday life **AND** The GP regards your joint pain as part of the normal ageing process that one just has to accept	0.02	35
B	The pain is disrupting some of your everyday life **AND** The GP regards your joint pain as a legitimate health problem that requires treatment	0.63	65
Scenario 2: Trade-off between available primary care assessment and management options vs perceived GP attitude			
A	The GP investigates with appropriate X-rays and blood tests as well as asks about your symptoms and their effect on your day-to-day life and conducts a thorough physical examination of the joints **AND** The GP offers a promising new treatment as well as prescribing pain relief, giving written advice about your condition and arranging follow-up with a practice nurse and physiotherapy referral **AND** The GP regards your joint pain as part of the normal ageing process that one just has to accept	−0.03	52
B	The GP asks about your symptoms and their effect on your day-to-day life **AND** The GP prescribes pain relief and gives verbal advice about your condition **AND** The GP regards your joint pain as a legitimate health problem that requires treatment	−0.10	48

*Within each pairwise scenario, the probability of choosing a profile (A or B) as the one under which they would be more likely to consult the GP (all else being equal). Calculated as the exponentiated total utility/sum total of exponentiated utilities.

GP, general practitioner.

##### Proposition 2: Changing to a (GP with a) positive legitimising GP attitude would encourage consultation more than the availability of thorough examination, investigations, new treatments and best-evidence management options

Almost half (48%) of respondents would opt to consult a GP with a ‘legitimising’ attitude offering basic assessment and management options (scenario A) than a GP offering a full range of investigations and treatments but who was perceived to have a ‘normal ageing-accept it’ attitude (scenario B; table 4).

## Discussion

Our conjoint analysis study confirms the importance of disability severity in determining the decision to consult for peripheral joint osteoarthritis but provides new quantitative evidence on the relative importance of perceived GP attitude. Anticipating an ‘it's normal ageing-accept it’ attitude was a strong disincentive to consulting having a stronger influence than intermittent, severe episodes of pain, competing comorbidities and the level of assessment and range of treatment options being offered. The majority of respondents indicated they would opt to consult a GP with a ‘legitimising’ attitude when experiencing less severe disability before they would visit a GP with ‘normal ageing-accept it’ when their disability was worse.

The clear association between degree of disrupted function and consultation for osteoarthritis is uncontentious and consistent with many previous studies.^19^ However, the relative importance of perceived doctor's attitude is novel and requires more careful interpretation. Access to healthcare can be considered through Wood *et al*^73^'s notion of ‘candidacy’, which refers to negotiation around an individual's eligibility for healthcare involving interaction between the health professional and patient, and which is influenced by cultural values.^74^ Legitimisation by the GP appears valued by patients and may be important to their perception as a good ‘candidate’ for consulting. Conversely a lack of legitimisation, whether experienced, perceived or anticipated, is likely to discourage consultation and the reporting of symptoms, a finding previously reported by McHugh *et al*^75^ and Haas^60^ and consistent with the importance of the endorsement and support of trusted primary healthcare professionals to accessing and adhering to arthritis self-management programmes.^76^ However, it is important not to over-interpret our findings. It must be borne in mind that our study does not provide evidence of the frequency with which persons with osteoarthritis feel their problem is not legitimised by their GP, merely that when this is the case it acts as a strong disincentive to consulting. A significant minority of participants in this study (17%) attributed their joint pain to ‘ageing’. Given that patients may see several different GPs, it would be useful to understand the extent to which negative expectations are transferred by patients from one practitioner to another.

Compared with previously published conjoint analysis studies in health,^77^ the present study was large and had a high response rate (although the sample frame comprised existing cohort participants). We involved members of the RUG through a series of meetings and qualitative developmental studies and believe this contributed to the response rate and low respondent burden. However, we did not use formal consensus development methods^78^ to derive the final list of attributes nor, given the strong advice from the RUG to use traditional pen and paper format, did we use computer-based adaptive conjoint analysis which would have enabled the initial inclusion of more attributes. It therefore remains possible that other, more powerful determinants of the decision to consult were not included in our study and therefore our findings must be interpreted in the context of those chosen attributes and specified levels. In addition, it is important to note that the estimated partworth utilities will reflect the particular attribute levels chosen and how these are framed. The partial-profile design, while minimising respondent burden, does not fully permit the estimation of interactions^65^ and thus our study is limited to estimating main effects only. In the evaluation of the pairwise scenarios, an assumption is made that the two variables, for example, legitimising attitude of GP and availability of investigations, are mutually exclusive. Although this is unlikely to fully reflect the inter-relationships in the real world, it does serve to demonstrate the relative value participants place on each variable. Finally, as with all such cross-sectional studies, our findings are a snapshot particular to time, place and person, and future research might usefully attempt to replicate these findings in a different setting.

Anticipating that the GP will regard joint pain as ‘part of the normal ageing process that one just has to accept’ is a strong disincentive to seeking help, potentially outweighing other aspects of quality of care (such as offering practice nurse follow-up and physiotherapy referral). Alongside the recognition and management of disrupted function, an important goal of each primary care consultation for osteoarthritis should be to avoid imparting or reinforcing this perception. Currently ongoing research studies within our Institution that could inform how this might be achieved include detailed, systematic observation of ‘negative talk’ within the osteoarthritis consultation,^76^ and an evaluation of the effects of implementing a ‘model OA consultation’ with patient guidebook in primary care.^79^”
